# Beyond conventional healthcare: public knowledge, attitudes, and associated demographic factors of complementary and alternative therapy utilization in the Qassim region, Saudi Arabia

**DOI:** 10.3389/fpubh.2026.1901544

**Published:** 2026-09-09

**Authors:** Shereen Ahmed A. Qalawa, Mohamed Goda Elbqry, Fatma Mohamed Elmansy, Lina Abdullah Alghofili, Sarah Saleh Alarifi, Moodhi Mohammad Alolayan, Rana Dhuwayhi Almutairi, Aljoud Abdulrahman Alshitwy, Buthainah Mohammed Alnoshan, Muneera Alharbi, Samia Eaid Elgazzar, Samah El-Awady Bassam, Abeer Yahia Mahdy Shalby, Amal Elhaj Alawad, Manal Th. Soliman

**Affiliations:** 1 Department of Medical-Surgical Nursing, College of Nursing, Qassim University, Buraydah, Saudi Arabia; 2 Department of Medical-Surgical Nursing, Faculty of Nursing, Port-Said University, Port-Said, Egypt; 3 Department of Nursing, Medical City, Qassim University, Buraydah, Saudi Arabia; 4 Department of Medical-Surgical Nursing, Faculty of Nursing, Suez Canal University, Ismailia, Egypt; 5 Department of Nursing, King Fahad Medical City, Minister of Health, Riyadh, Saudi Arabia; 6 Department of Nursing, Buraidah Central Hospital, Minister of Health, Buraydah, Saudi Arabia; 7 Department of Nursing, Al Midhnab General Hospital, Minister of Health, Al Midhnab, Saudi Arabia; 8 Department of Maternal and Child Health, College of Nursing, Qassim University, Buraydah, Saudi Arabia; 9 Department of Pediatric Nursing, Faculty of Nursing, Zagazig University, Zagazig, Egypt; 10 Department of Medical-Surgical Nursing, College of Nursing, Najran University, Najran, Egypt; 11 Department of Medical-Surgical Nursing, Faculty of Nursing, Banha University, Banha, Egypt; 12 Department of Microbiology, Collage of Medicine, Najran University, Najran City, Saudi Arabia; 13 Department of Medical-Surgical Nursing, Mansoura University, Mansoura, Egypt; 14 Critical Care and Emergency Nursing Department, Faculty of Nursing, University of Hafr Albatin, Hafr Albatin, Saudi Arabia

**Keywords:** alternative therapy, attitudes, complementary therapy, demographic factors, knowledge, public health, Qassim region, Saudi Arabia

## Abstract

**Background:**

Complementary and alternative therapies (CAT) are increasingly used worldwide as adjuncts to conventional healthcare. Understanding public knowledge, attitudes, and utilization patterns of CAT is essential for promoting safe and informed healthcare decision-making. However, evidence regarding public perceptions and demographic factors associated with CAT utilization in Saudi Arabia remains limited.

**Aim:**

This study aimed to assess public knowledge and attitudes toward complementary and alternative therapies (CAT), determine the prevalence of CAT utilization, and identify the demographic factors associated with CAT utilization among adults in the Qassim Region, Saudi Arabia.

**Methods:**

A community-based cross-sectional survey was conducted among adults residing in the Qassim Region, Saudi Arabia. Data were collected using a structured online questionnaire assessing participants’ socio-demographic characteristics, knowledge, attitudes, and utilization of CAT. Descriptive and inferential statistical analyses were performed to examine factors associated with CAT utilization.

**Results:**

A total of 1,586 participants were included in the study, the majority of whom were female (82.0%) and Saudi nationals (97.1%). Nearly half of the participants were students (48.5%), and 66.1% reported having adequate income. Overall, 62.3% of participants demonstrated adequate knowledge regarding CAT, while 70.1% exhibited positive attitudes toward CAT. More than half of the participants (51.3%) reported previous or current utilization of CAT. No significant differences in CAT utilization were observed according to gender or nationality. However, occupation, income level, marital status, and educational attainment were significantly associated with CAT utilization (*p* < 0.05).

**Conclusion:**

Complementary and alternative therapies are widely recognized and utilized among adults in the Qassim Region. Participants generally demonstrated adequate knowledge and positive attitudes toward CAT; however, utilization patterns varied according to several demographic characteristics, particularly occupation, income, marital status, and educational level. These findings highlight the need for targeted public health education programs to promote evidence-based CAT use, improve awareness of potential risks and adverse effects, and support informed healthcare decision-making.

## Introduction

Complementary and alternative therapies (CAT) have gained increasing popularity worldwide as adjuncts to conventional healthcare (1). These therapies encompass a broad range of health practices, approaches, and products that are not traditionally considered part of mainstream medicine but are used to promote health, prevent disease, and manage various physical and psychological conditions (2). CAT includes biologically based approaches such as herbal medicine, dietary supplements, vitamins, and nutritional products, as well as non-biological modalities including acupuncture, massage therapy, hydrotherapy, cupping therapy (hijama), music therapy, and spiritual healing (3, 4).

The growing utilization of CAT has been attributed to increased public interest in holistic healthcare, concerns regarding adverse effects associated with conventional treatments, and a desire for greater participation in personal health management (5, 6). The World Health Organization recognizes traditional, complementary, and integrative medicine as an important component of healthcare systems and highlights its potential contribution to disease prevention, health promotion, and the management of chronic conditions (7). Consequently, CAT is increasingly used worldwide for the management of chronic diseases such as diabetes mellitus, hypertension, arthritis, cardiovascular disorders, neurological conditions, chronic pain, anxiety, stress, and sleep disturbances (8, 9).

In Saudi Arabia, CAT utilization is strongly influenced by cultural traditions, religious beliefs, and long-standing therapeutic practices (10). Herbal medicine, cupping therapy, spiritual healing, dietary supplements, and other traditional remedies are commonly used either as alternatives or complements to conventional medical treatments (11). Many individuals perceive these therapies as natural, culturally acceptable, and associated with fewer side effects than conventional medicine. As a result, CAT has become an increasingly important component of healthcare-seeking behavior among the Saudi population, reflecting a growing interest in holistic and patient-centered approaches to health and well-being (12).

Despite its widespread use, concerns remain regarding the safety, effectiveness, and appropriate utilization of CAT. Certain therapies may interact with prescribed medications, delay diagnosis and treatment, or contribute to adverse health outcomes when used without professional supervision (13). Herbal products and dietary supplements, in particular, may result in clinically significant drug–herb interactions or unexpected side effects (14). Therefore, improving public awareness regarding the benefits, limitations, and potential risks of CAT is essential to support informed healthcare decisions and promote patient safety.

Healthcare professionals, particularly nurses, play a critical role in educating patients about the safe and evidence-based use of CAT (15). Through patient education, counseling, and health promotion activities, nurses can facilitate informed decision-making, encourage disclosure of CAT use, and support the integration of appropriate complementary therapies within conventional healthcare settings (16).

**Figure 1:**
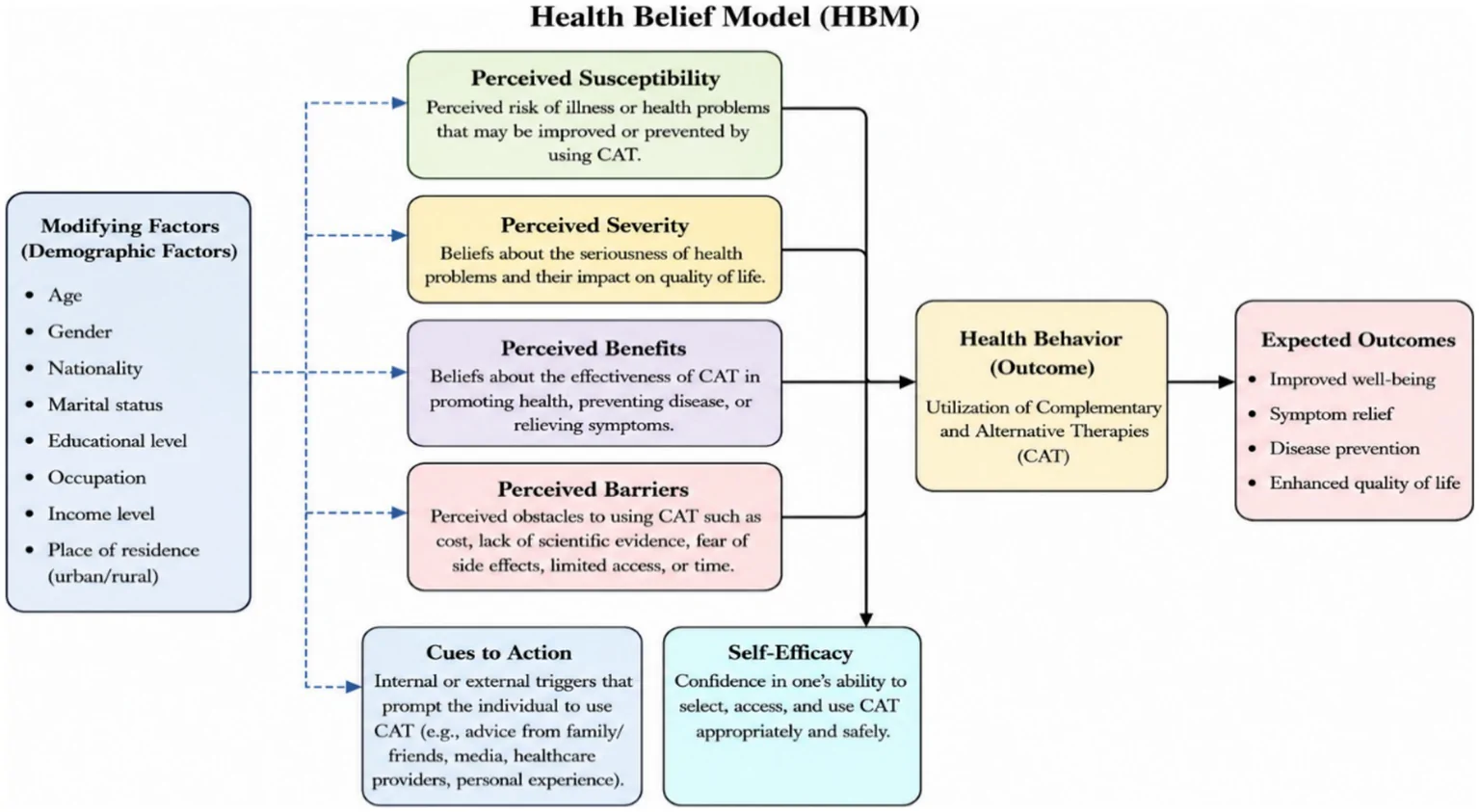
Conceptual framework of the health belief model (HBM) applied for complementary and alternative (CAT) therapies.

The present study was informed by the Health Belief Model (HBM), a widely recognized theoretical framework for understanding health-related behaviors (Figure 1). The HBM proposes that individuals’ healthcare decisions are influenced by their perceptions of the benefits and barriers associated with health behavior, as well as their beliefs regarding health outcomes and available treatment options (17). In the context of CAT, individuals with greater knowledge and positive attitudes toward these therapies may be more likely to utilize them. Furthermore, demographic characteristics such as age, gender, educational level, marital status, occupation, and income may influence health beliefs and healthcare-seeking behaviors, thereby affecting CAT utilization. HBM therefore provides a useful framework for examining the relationships among knowledge, attitudes, demographic factors, and utilization of CAT (18).

Although previous studies have reported increasing utilization of CAT in different populations, evidence regarding public knowledge, attitudes, and utilization patterns remains limited in Saudi Arabia, particularly at the regional level. Furthermore, little is known about the demographic factors associated with CAT utilization among adults residing in the Qassim Region. Understanding these factors is essential for developing targeted educational interventions, informing healthcare policies, and promoting the safe and evidence-based integration of CAT into healthcare practice. Therefore, this study aimed to assess public knowledge, attitudes, utilization, and associated demographic factors related to complementary and alternative therapies among adults in the Qassim Region, Saudi Arabia.

## Aim of study

This study aimed to assess public knowledge and attitudes toward complementary and alternative therapies (CAT), determine the prevalence of CAT utilization, and identify the demographic factors associated with CAT utilization among adults in the Qassim Region, Saudi Arabia.

Research questions

1. What is the prevalence of complementary and alternative therapy (CAT) utilization among adults in the Qassim Region, Saudi Arabia?
2. What is the level of knowledge regarding complementary and alternative therapies among adults in the Qassim Region, Saudi Arabia?
3. What are the attitudes of adults toward complementary and alternative therapies in the Qassim Region, Saudi Arabia?
4. Are socio-demographic characteristics (e.g., age, gender, nationality, occupation, monthly income, marital status, educational level, and religion) associated with complementary and alternative therapy utilization?
5. Are socio-demographic characteristics associated with participants’ knowledge and attitudes regarding complementary and alternative therapies?
6. Is there an association between participants’ knowledge and attitudes regarding complementary and alternative therapies and their utilization of these therapies?

## Methods

### Study design and setting

A community-based cross-sectional survey study was conducted among adults residing in the Qassim Region, Saudi Arabia, between March and June 2024. The study targeted community-dwelling adults from various cities and governorates within the region.

### Participants and sampling

A convenience sampling technique was employed to recruit eligible participants from the Qassim Region, Saudi Arabia. Adults who were residents of the Qassim region, able to read and willing to participate were invited to complete the survey. Individuals who declined participation, submitted incomplete questionnaires, or reported cognitive impairments that could affect their ability to understand and respond to the survey were excluded. Participation was entirely voluntary, and electronic informed consent was obtained from all participants prior to survey completion.

The required sample size was estimated using Cochran’s formula for cross-sectional studies with a 95% confidence level, a 5% margin of error, and an assumed population proportion of 50%, yielding a minimum required sample of 384 participants. To improve the precision and reliability of the study findings and increase statistical power, a relatively substantially sample was recruited. A total of 1,586 participants completed the survey and were included in the final analysis, representing more than four times the minimum required sample size and providing adequate statistical power for all planned analyses.

### Data collection tools

Data were collected using a structured, self-administered questionnaire online survey adapted from previous related studies (9, 10). The questionnaire was designed to assess participants’ demographic characteristics, ACT Utilization, knowledge, attitudes, and associated demographic factors. The instrument consisted of three sections. The original questionnaire was developed in English and translated into Arabic using the forward–backward translation technique to ensure linguistic and cultural equivalence. Initially, the English version was translated into Arabic by two independent bilingual experts. Subsequently, the Arabic version was back-translated into English by an independent bilingual translator who was blinded to the original instrument. The translated versions were reviewed and compared, and any discrepancies were resolved through discussion among the research team to ensure conceptual accuracy, semantic equivalence, and cultural appropriateness for the target population.

#### Section I: demographic characteristics questionnaire

This section was developed by the researchers and consisted of nine items assessing participants’ socio-demographic characteristics, including age, gender, nationality, place of residence, occupation, monthly income, marital status, educational level, and religion.

#### Section II: knowledge regarding complementary and alternative therapies

This section consisted of 12 items designed to assess participants’ knowledge of common complementary and alternative therapy (CAT) modalities, including herbal medicine, spiritual healing, massage therapy, nutritional supplements, and cupping therapy (hijama). Each correct response was assigned one point, while incorrect or “do not know” responses received zero points, yielding a total knowledge score ranging from 0 to 12. The overall knowledge score was calculated by summing the responses to all 12 items and converting the total score into a percentage. Participants scoring 8–12 points (≥60%) were classified as having adequate knowledge, whereas those scoring 0–7 points (<60%) were classified as having inadequate knowledge (9).

#### Section III: attitudes toward complementary and alternative therapies

This section consisted of six items assessing participants’ attitudes toward CAT, including perceptions of effectiveness, safety, integration with conventional medicine, consultation preferences, willingness to recommend CAT to others, and perceived cost-effectiveness. Responses were measured using a five-point Likert scale ranging from strongly disagree (1) to strongly agree (5). Total attitude scores were converted into percentages, with scores ≥60% indicating a positive attitude toward CAT and scores <60% indicating a negative attitude (10).

### Pilot study

A pilot study was conducted on 10% of the estimated sample size to evaluate the clarity, feasibility, readability, and estimated completion time of the questionnaire. Participants included in the pilot study were excluded from the final analysis. Feedback obtained from the pilot participants resulted in minor modifications to the wording and sequence of selected items to improve clarity and comprehension.

### Validity

Content validity was established by a panel of five experts in nursing, public health, and complementary medicine who evaluated the questionnaire for clarity, relevance, comprehensiveness, and cultural appropriateness. Based on their feedback, minor revisions were made to improve the wording and organization of selected items. The overall Content Validity Index (CVI) of the instrument was 0.92, indicating excellent content validity according to the recommended criteria (9).

### Reliability

Reliability testing was conducted using Cronbach’s alpha coefficient to assess internal consistency. The knowledge and attitude scales demonstrated Cronbach’s alpha coefficients of 0.79 and 0.80, respectively, indicating acceptable internal consistency of the study instrument according to the recommended threshold of α ≥ 0.70 (10).

### Data collection procedure

Data were collected between March and June 2024 using a secure online questionnaire created through Microsoft Forms. The survey link and Quick Response (QR) code were disseminated through various social media platforms, including social WhatsApp, Telegram, and Snapchat, as well as community networks across different cities and governorates of the Qassim Region, Saudi Arabia. This distribution strategy facilitated broad community outreach and enhanced participation from diverse demographic groups. Prior to accessing the questionnaire, potential participants were provided with an electronic information sheet explaining the study objectives, procedures, voluntary nature of participation, confidentiality measures, and participants’ rights.

Electronic informed consent was obtained before participants could proceed with the survey. The average time required to complete the questionnaire ranged from 3 to 5 min. To minimize duplicate submissions, the questionnaire settings were configured to allow only one response per participant. Responses were reviewed for completeness and eligibility, and incomplete questionnaires were excluded from the final analysis. A total of 1,586 completed questionnaires met the eligibility criteria and were included in the study. All data were automatically stored in a secure password-protected database accessible only to the research team and were used exclusively for research purposes.

### Statistical design

Data were analyzed using the Statistical Package for the Social Sciences (SPSS) version 27.0 (IBM Corp., Armonk, NY, United States). Prior to analysis, data were checked for completeness, accuracy, and normality. Descriptive statistics were used to summarize the study variables. Categorical variables were presented as frequencies and percentages, whereas continuous variables were presented as means and standard deviations (Mean ± SD). Knowledge and attitude scores were calculated according to the predefined scoring system and categorized into adequate/inadequate knowledge and positive/negative attitudes, respectively.

Inferential statistical analyses were conducted to examine associations between study variables. The Chi-square (*χ*^2^) test was used to assess associations between categorical variables, including the relationships between socio-demographic characteristics, knowledge level, attitude level, and complementary and alternative therapy (CAT) utilization. Crude odds ratios (ORs) with 95% confidence intervals (CIs) were calculated where appropriate. Variables that demonstrated statistical significance in the bivariate analyses were subsequently entered into a multivariable binary logistic regression model to identify independent predictors of CAT utilization. Adjusted odds ratios (AORs) with 95% confidence intervals (CIs) were reported. Model fitness was assessed using the Hosmer–Lemeshow goodness-of-fit test and Nagelkerke’s R^2^. A two-tailed *p*-value of less than 0.05 was considered statistically significant throughout the analysis.

## Results

Table 1 presents the mean age of the participants was 45.5 ± 6.9 years. The majority of participants were female (82.0%) and Saudi nationals (97.1%). Nearly half of the participants were students (48.5%), while 27.7% were employed. Most participants reported having adequate monthly income (66.1%) and were single (60.8%). Regarding educational attainment, approximately two-thirds of the participants held a university degree (63.7%), followed by a high school education (19.1%). Almost all participants identified as Muslim (99.8%). Out of 1,586 participants, 814 participants reported previous or current use of CAT, representing 51.3%, while 772 participants reported no CAT use, representing 48.7%.

**Table 1:** Socio-demographic characteristics of the study participants (*N* = 1,586).

Variable	Category	*n*	%
Age (years) Mean ± SD (45.5 ± 6.9)			
Gender	Male	286	18.0
	Female	1,300	82.0
Nationality	Saudi	1,540	97.1
	Non-Saudi	46	2.9
Occupation	Student	769	48.5
	Employee	440	27.7
	Unemployed	125	7.9
	Retired	102	6.4
	Self-employed	133	8.4
	Housewife	17	1.1
Monthly income	Inadequate	538	33.9
	Adequate	1,048	66.1
Marital status	Single	965	60.8
	Married	565	35.6
	Widowed	20	1.3
	Divorced	36	2.3
Educational level	Unformal education	1	0.1
	Read and write	9	0.6
	Primary school	11	0.7
	Middle school	26	1.6
	High school	303	19.1
	Diploma	158	10.0
	University degree	1,010	63.7
	Postgraduate degree	68	4.3
Religion	Muslim	1,583	99.8
	Non-Muslim	3	0.2
Have you ever utilized complementary and alternative therapy?	Yes	814	51.3
	No	772	48.7

*n*, frequency.

Table 1 presents the socio-demographic characteristics of the 1,586 study participants.

Table 2 presents participants’ knowledge regarding complementary and alternative therapies (CAT). Most participants reported having information about modern medical treatments (72.3%) and CAT (62.3%). Awareness of common CAT modalities was generally high, particularly for cupping therapy (89.0%), spiritual healing (86.9%), herbal medicine (83.5%), and massage therapy (79.2%). Approximately two-thirds of participants recognized the importance of consulting healthcare professionals before using CAT (69.9%) and understood that CAT should not replace conventional medical treatment without professional advice (65.1%). However, awareness of potential side effects associated with CAT (40.2%) and knowledge regarding the scientific evidence supporting some CAT modalities (54.5%) were comparatively lower. Overall, 62.3% of participants demonstrated adequate knowledge regarding CAT, whereas 37.7% had inadequate knowledge.

**Table 2:** Participants’ knowledge regarding complementary and alternative therapies (*N* = 1,586).

Knowledge items		Frequency	
		Yes *n* (%)	No *n* (%)
Have information about modern medical treatments (medications, surgeries, and vaccines).		1,146 (72.3)	440 (27.7)
Have general information about complementary and alternative therapies (CAT).		988 (62.3)	598 (37.7)
Know the potential side effects of CAT.		638 (40.2)	948 (59.8)
Know that herbal products may interact with prescribed medications.		921 (58.1)	665 (41.9)
Know that CAT should not replace conventional medical treatment.		1,032 (65.1)	554 (34.9)
Knowing the importance of consulting before using CAT.		1,108 (69.9)	478 (30.1)
Have heard about herbal medicine as a CAT modality.		1,324 (83.5)	262 (16.5)
Have heard about cupping therapy (hijama) as a CAT modality.		1,412 (89.0)	174 (11.0)
Have heard about massage therapy as a CAT modality.		1,256 (79.2)	330 (20.8)
Have heard about spiritual healing as a CAT modality.		1,378 (86.9)	208 (13.1)
Know that some CAT modalities are supported by scientific evidence.		864 (54.5)	722 (45.5)
Know that improper use of CAT may lead to adverse health outcomes.		1,004 (63.3)	582 (36.7)
Overall, knowledge level	Adequate knowledge	988	62.3
	Inadequate knowledge	598	37.7

CAT, Complementary and alternative therapies.

Table 3 presents participants’ attitudes toward complementary and alternative therapies (CAT). Nearly half of the participants agreed or strongly agreed that CAT should be integrated with conventional medicine (49.4%), reflecting support for a complementary approach to healthcare. Similarly, 48.5% perceived CAT as less expensive than conventional medication. Approximately one-third of participants agreed that CAT is safer than conventional medication (33.2%). In contrast, most participants did not prefer consulting CAT practitioners before physicians (53.9%) and were unlikely to recommend visiting CAT practitioners as the first option for treatment (52.7%). Overall, 70.1% of participants demonstrated a positive attitude toward CAT, whereas 29.9% exhibited a negative attitude, indicating broad acceptance of CAT as a complementary component of healthcare rather than a substitute for conventional medical treatment.

**Table 3:** Participants’ attitudes toward complementary and alternative therapies (CAT) (*N* = 1,586).

Attitude items		Strongly disagree *n* (%)	Disagree *n* (%)	Neutral *n* (%)	Agree *n* (%)	Strongly agree *n* (%)
Complementary and alternative therapy is more effective than conventional medication		88 (5.5)	328 (20.7)	851 (53.7)	242 (15.3)	77 (4.9)
Complementary and alternative therapy is safer than conventional medication		90 (5.7)	304 (19.2)	665 (41.9)	374 (23.6)	153 (9.6)
Complementary and alternative therapy should be integrated with conventional medicine		102 (6.4)	235 (14.8)	465 (29.3)	540 (34.0)	244 (15.4)
I prefer consulting complementary and alternative therapy practitioners before physicians		304 (19.2)	550 (34.7)	367 (23.1)	247 (15.6)	118 (7.4)
I would recommend visiting complementary and alternative therapy practitioners before physicians		287 (18.1)	549 (34.6)	414 (26.1)	245 (15.4)	91 (5.7)
Complementary and alternative therapy is less expensive than conventional medication		71 (4.5)	209 (13.2)	537 (33.9)	547 (34.5)	222 (14.0)
Overall attitude level	Positive attitude	1,112 (70.1)				
	Negative attitude	474 (29.)				

CAT, Complementary and alternative therapies.

Table 4 presents the association between participants’ socio-demographic characteristics and complementary and alternative therapy (CAT) utilization. No statistically significant differences in CAT utilization were observed according to gender (*p* = 0.072) or nationality (*p* = 0.630). However, occupation was significantly associated with CAT utilization (*p* < 0.001), with the highest utilization reported among self-employed participants (72.9%) and retired individuals (68.6%). Monthly income was also significantly associated with CAT utilization (*p* = 0.010), as participants with adequate income reported higher CAT use (53.6%) than those with inadequate income (46.8%).

**Table 4:** Association between participants’ socio-demographic characteristics and complementary and alternative therapy (CAT) utilization (*N* = 1,586).

Socio-demographic characteristics	Category	CAT non-users *n* (%)	CAT users *n* (%)	*p*-value
Gender	Male	153 (53.5)	133 (46.5)	0.072
	Female	619 (47.6)	681 (52.4)	
Nationality	Saudi	748 (48.6)	792 (51.4)	0.630
	Non-Saudi	24 (52.2)	22 (47.8)	
Occupation	Student	461 (59.9)	308 (40.1)	<0.001*
	Employee	172 (39.1)	268 (60.9)	
	Unemployed	62 (49.6)	63 (50.4)	
	Retired	32 (31.4)	70 (68.6)	
	Self-employed	36 (27.1)	97 (72.9)	
	Housewife	9 (52.9)	8 (47.1)	
Monthly income	Inadequate	286 (53.2)	252 (46.8)	0.010*
	Adequate	486 (46.4)	562 (53.6)	
Marital status	Single	569 (59.0)	396 (41.0)	<0.001*
	Married	185 (32.7)	380 (67.3)	
	Widowed	6 (30.0)	14 (70.0)	
	Divorced	12 (33.3)	24 (66.7)	
Educational level	Unformal education	0 (0.0)	1 (100.0)	0.002*
	Read and write	6 (66.7)	3 (33.3)	
	Primary school	5 (45.5)	6 (54.5)	
	Middle school	12 (46.2)	14 (53.8)	
	High school	173 (57.1)	130 (42.9)	
	Diploma	57 (36.1)	101 (63.9)	
	University degree	491 (48.6)	519 (51.4)	
	Postgraduate degree	28 (41.2)	40 (58.8)	

Statistically significant at *p* < 0.05.

Marital status demonstrated a significant association with CAT utilization (*p* < 0.001). Higher utilization rates were observed among widowed (70.0%), married (67.3%), and divorced (66.7%) participants compared with single individuals (41.0%). Educational level was likewise significantly associated with CAT utilization (*p* = 0.002), with higher utilization reported among participants holding diploma (63.9%), postgraduate (58.8%), and university degrees (51.4%). Overall, occupation, monthly income, marital status, and educational level were significant demographic correlates of CAT utilization, whereas gender and nationality were not significantly associated with CAT use.

Table 5 presents the association between participants’ socio-demographic characteristics and knowledge level regarding complementary and alternative therapies (CAT). Female participants demonstrated significantly higher levels of adequate knowledge than males (63.6% vs. 56.3%, *p* = 0.021). Knowledge level was significantly associated with occupation (*p* < 0.001), with the highest levels of adequate knowledge observed among self-employed (76.7%) and retired participants (76.5%). Participants with adequate monthly income were more likely to demonstrate adequate knowledge than those with inadequate income (64.7% vs. 57.6%, *p* = 0.008). Marital status was also significantly associated with knowledge level (*p* < 0.001), with married and widowed participants exhibiting higher levels of adequate knowledge than single and divorced individuals. Furthermore, educational level showed a significant association with knowledge level (*p* < 0.001), as participants with diploma, university, and postgraduate education demonstrated higher levels of adequate knowledge regarding CAT. No significant association was observed between nationality and knowledge level (*p* = 0.641).

**Table 5:** Association between participants’ socio-demographic characteristics and knowledge level regarding complementary and alternative therapies (CAT) (*N* = 1,586).

Socio-demographic characteristics	Category	Inadequate knowledge *n* (%)	Adequate knowledge *n* (%)	*p*-value
Gender	Male	125 (43.7)	161 (56.3)	0.021*
	Female	473 (36.4)	827 (63.6)	
Nationality	Saudi	578 (37.5)	962 (62.5)	0.641
	Non-Saudi	20 (43.5)	26 (56.5)	
Occupation	Student	351 (45.6)	418 (54.4)	<0.001*
	Employee	132 (30.0)	308 (70.0)	
	Unemployed	52 (41.6)	73 (58.4)	
	Retired	24 (23.5)	78 (76.5)	
	Self-employed	31 (23.3)	102 (76.7)	
	Housewife	8 (47.1)	9 (52.9)	
Monthly income	Inadequate	228 (42.4)	310 (57.6)	0.008*
	Adequate	370 (35.3)	678 (64.7)	
Marital status	Single	421 (43.6)	544 (56.4)	<0.001*
	Married	151 (26.7)	414 (73.3)	
	Widowed	5 (25.0)	15 (75.0)	
	Divorced	21 (58.3)	15 (41.7)	
Educational level	Unformal education	1 (100.0)	0 (0.0)	<0.001*
	Read and write	7 (77.8)	2 (22.2)	
	Primary school	8 (72.7)	3 (27.3)	
	Middle school	15 (57.7)	11 (42.3)	
	High school	161 (53.1)	142 (46.9)	
	Diploma	46 (29.1)	112 (70.9)	
	University degree	334 (33.1)	676 (66.9)	
	Postgraduate degree	26 (38.2)	42 (61.8)	

CAT, Complementary and alternative therapies. Statistically significant at *p* < 0.05.

Table 6 demonstrates the association between participants’ socio-demographic characteristics and their attitudes toward complementary and alternative therapies (CAT). Overall, 70.1% of participants exhibited a positive attitude toward CAT, whereas 29.9% demonstrated a negative attitude. No statistically significant associations were observed between attitude and gender (*p* = 0.158), nationality (*p* = 0.765), or monthly income (*p* = 0.548). In contrast, occupation (*p* < 0.001), marital status (*p* < 0.001), and educational level (*p* = 0.001) were significantly associated with participants’ attitudes toward CAT. Positive attitudes were generally more prevalent among employees, retired individuals, married participants, and those with higher educational attainment, whereas students demonstrated comparatively lower positive attitudes.

**Table 6:** Association between participants’ socio-demographic characteristics and attitudes toward complementary and alternative therapies (CAT) (*N* = 1,586).

Socio-demographic characteristics	Category	Negative attitude *n* (%)	Positive attitude *n* (%)	*p*-value
Gender	Male	86 (30.1)	200 (69.9)	0.158
	Female	388 (29.8)	912 (70.2)	
Nationality	Saudi	460 (29.9)	1,080 (70.1)	0.765
	Non-Saudi	14 (30.4)	32 (69.6)	
Occupation	Student	300 (39.0)	469 (61.0)	<0.001*
	Employee	80 (18.2)	360 (81.8)	
	Unemployed	30 (24.0)	95 (76.0)	
	Retired	20 (19.6)	82 (80.4)	
	Self-employed	40 (30.1)	93 (69.9)	
	Housewife	4 (23.5)	13 (76.5)	
Monthly income	Inadequate	189 (35.1)	349 (64.9)	0.548
	Adequate	285 (27.2)	763 (72.8)	
Marital status	Single	335 (34.7)	630 (65.3)	<0.001*
	Married	124 (22.0)	441 (78.0)	
	Widowed	4 (20.0)	16 (80.0)	
	Divorced	11 (30.6)	25 (69.4)	
Educational level	Unformal education	1 (100.0)	0 (0.0)	0.001*
	Read and write	2 (22.2)	7 (77.8)	
	Primary school	3 (27.3)	8 (72.7)	
	Middle school	6 (23.1)	20 (76.9)	
	High school	92 (30.4)	211 (69.6)	
	Diploma	39 (24.7)	119 (75.3)	
	University degree	319 (31.6)	691 (68.4)	
	Postgraduate degree	12 (17.6)	56 (82.4)	

CAT, Complementary and alternative therapies. Statistically significant at *p* < 0.05.

Table 7 presents the association between participants’ knowledge level, attitude level, and complementary and alternative therapy (CAT) utilization. A statistically significant association was observed between knowledge level and CAT utilization (*p* < 0.001). Participants with adequate knowledge were more likely to report CAT utilization (56.5%) compared with those with inadequate knowledge (42.8%). Similarly, attitude level was significantly associated with CAT utilization (*p* < 0.001). Participants with positive attitudes toward CAT demonstrated higher utilization rates (56.1%) than those with negative attitudes (40.1%). These findings suggest that both adequate knowledge and positive attitudes are important factors associated with the utilization of complementary and alternative therapies.

**Table 7:** Association between knowledge level, attitude level, and complementary and alternative therapy (CAT) utilization among participants (*N* = 1,586).

Variable	Category	CAT non-users *n* (%)	CAT users *n* (%)	*p*-value
Knowledge level	Inadequate	342 (57.2)	256 (42.8)	<0.001*
	Adequate	430 (43.5)	558 (56.5)	
Attitude level	Negative	284 (59.9)	190 (40.1)	<0.001*
	Positive	488 (43.9)	624 (56.1)	

CAT, Complementary and alternative therapies. Statistically significant at *p* < 0.05.

Table 8 presents the multivariable logistic regression analysis of factors associated with complementary and alternative therapy (CAT) utilization. After adjusting for potential confounders, participants with adequate knowledge were significantly more likely to utilize CAT than those with inadequate knowledge (AOR = 1.82, 95% CI: 1.45–2.29, *p* < 0.001). Similarly, participants with positive attitudes toward CAT had significantly higher odds of CAT utilization (AOR = 1.67, 95% CI: 1.31–2.12, *p* < 0.001). Compared with students, employees, retired, and self-employed participants were significantly more likely to utilize CAT, whereas no significant association was observed among unemployed participants or housewives. Participants with adequate monthly income were more likely to use CAT than those with inadequate income (AOR = 1.29, 95% CI: 1.04–1.61, *p* = 0.021). Compared with single participants, married and widowed participants demonstrated significantly higher odds of CAT utilization, while no significant association was found for divorced participants. Regarding educational level, participants with a diploma or university degree were significantly more likely to utilize CAT than those with a high school education, whereas no significant associations were observed for unformal education, read-and-write literacy, primary school, middle school, or postgraduate education.

**Table 8:** Multivariable logistic regression analysis of factors associated with complementary and alternative therapy (CAT) utilization among adults in the Qassim Region (*N* = 1,586).

Predictor variables	Category	AOR	95% CI	*p*-value
Knowledge level	Inadequate (Ref.)	—	—	—
	Adequate	1.82	1.45–2.29	<0.001*
Attitude level	Negative (Ref.)	—	—	—
	Positive	1.67	1.31–2.12	<0.001*
Occupation	Student (Ref.)	—	—	—
	Employee	1.58	1.21–2.05	0.001*
	Unemployed	1.20	0.82–1.74	0.418
	Retired	2.43	1.51–3.92	<0.001*
	Self-employed	2.76	1.84–4.15	<0.001*
	Housewife	1.27	0.74–2.19	0.502
Monthly income	Inadequate (Ref.)	—	—	—
	Adequate	1.29	1.04–1.61	0.021*
Marital status	Single (Ref.)	—	—	—
	Married	1.74	1.38–2.20	<0.001*
	Widowed	2.01	1.02–3.98	0.044*
	Divorced	1.43	0.88–2.33	0.147
Educational level	High school (Ref.)	—	—	—
	Unformal education	0.81	0.09–7.28	0.851
	Read & write	1.08	0.29–4.06	0.912
	Primary school	1.16	0.40–3.36	0.782
	Middle school	1.24	0.58–2.67	0.561
	Diploma	1.52	1.09–2.13	0.014*
	University degree	1.36	1.04–1.79	0.026*
	Postgraduate degree	1.41	0.89–2.24	0.142

All variables of interest were entered simultaneously into the multivariable logistic regression model using the enter method. Reference categories included inadequate knowledge, negative attitude, student (occupation), inadequate monthly income, single marital status, and high school educational level. Model fit was assessed using the Hosmer–Lemeshow goodness-of-fit test (*χ*^2^ = 6.18, *p* = 0.627), indicating adequate model fit. The model explained 24.3% of the variance in CAT utilization (Nagelkerke *R*^2^ = 0.243) and was statistically significant overall (*χ*^2^ = 124.57, *p* < 0.001). AOR, Adjusted Odds Ratio; CI, Confidence Interval. Statistically significant at *p* < 0.05.

## Discussion

Complementary and alternative therapies (CAT) have become increasingly recognized as important components of healthcare worldwide and within Saudi Arabia (2–10). The growing interest in CAT reflects increasing public demand for holistic approaches that address physical, psychological, social, and spiritual aspects of health. The present study assessed public knowledge, attitudes, and utilization of CAT among adults in the Qassim Region and explored the demographic factors associated with CAT utilization. The findings provide important insights into the current status of CAT awareness, perceptions, and use within the Saudi community.

One of the principal findings of this study was that 51.3% of participants reported previous or current utilization of CAT. This prevalence is comparable to the lifetime prevalence reported by Posadzki et al. (51.8%) among the general population in the United Kingdom (15). It also falls within the broad range documented in systematic reviews, which reported utilization rates ranging from 9 to 65% (16) and from 9.8 to 76% (17). Within Saudi Arabia, previous studies have likewise reported substantial utilization of CAT among community populations (14–18). The prevalence identified in the current study therefore falls within the range reported nationally and internationally. This widespread utilization may be attributed to the cultural acceptance of traditional healing practices, religious beliefs, and increasing public interest in preventive and holistic healthcare approaches.

The present study revealed that 62.3% of participants demonstrated adequate knowledge regarding CAT. Participants showed particularly high awareness of commonly used therapies such as cupping therapy, spiritual healing, herbal medicine, and massage therapy. This finding is consistent with previous studies conducted among Saudi populations and healthcare students, which reported moderate-to-high levels of awareness regarding CAT and traditional healing practices (2–19). Similarly, Belachew et al. (9) reported that community residents with greater exposure to health information demonstrated higher levels of CAT knowledge. The relatively high level of knowledge observed in the current study may be explained by the widespread accessibility of health information through digital platforms and the cultural familiarity of the Saudi population with traditional therapeutic practices.

However, the current findings differ from studies conducted among healthcare and university students, which reported lower levels of knowledge regarding the indications, effectiveness, and safety of CAT therapies (20, 21). These discrepancies may be attributed to differences in study populations, educational exposure, cultural background, and accessibility of CAT-related information. Despite the generally adequate level of knowledge observed in the present study, only 40.2% of participants reported awareness of the potential side effects associated with CAT use. This finding suggests that public understanding may be focused primarily on perceived benefits rather than potential risks. Similar concerns have been reported by Bello et al. (13), who emphasized inadequate awareness regarding adverse effects, herb–drug interactions, and the importance of discussing CAT use with healthcare providers.

Attitudes toward CAT were generally favorable, with 70.1% of participants demonstrating a positive attitude. Nearly half of the respondents supported integrating CAT with conventional medical treatment, reflecting increasing acceptance of integrative healthcare models. These findings are consistent with studies conducted among healthcare students and community populations that reported positive perceptions and acceptance of CAT as a complementary healthcare approach (2–7). Likewise, healthcare professionals in Saudi Arabia generally recognized the potential value of complementary therapies in patient care (1). Similar positive attitudes were documented among university students in Pakistan, Kuwait, Sierra Leone, and Ghana (19–22).

Positive attitudes toward CAT may stem from perceptions that these therapies are natural, culturally acceptable, and capable of improving overall well-being. However, the current findings differ from studies conducted in some educational settings, where participants expressed more cautious or neutral attitudes toward CAT because of concerns regarding limited scientific evidence, insufficient regulation, and uncertainty about safety and effectiveness (23, 24). Such concerns may explain why many participants in the present study preferred consulting physicians before CAT practitioners despite expressing generally positive attitudes toward CAT. This observation suggests that participants viewed CAT as complementary rather than replacement therapy, a finding that is consistent with previous reports (3–25).

The internet and social media emerged as major sources of information regarding CAT. This finding is consistent with the review conducted by Ng et al. (24), which highlighted the increasing role of social media platforms in disseminating information related to complementary and alternative medicine. While digital platforms facilitate access to health information, they may also contribute to the spread of misinformation regarding the effectiveness and safety of CAT. Therefore, healthcare organizations and public health authorities should actively engage in providing reliable and evidence-based information through these communication channels.

The current findings also support the applicability of the Health Belief Model in explaining CAT-related behaviors. Participants with greater knowledge and more favorable attitudes were significantly more likely to utilize CAT. This interpretation is supported by Dehghan et al. (8), who reported a positive relationship between health literacy and complementary medicine use among patients with chronic illnesses. Individuals who perceive CAT as beneficial, accessible, and culturally acceptable may therefore be more inclined to adopt these therapies.

The present study further demonstrated significant associations between knowledge level, attitude level, and CAT utilization. Participants with adequate knowledge and positive attitudes were significantly more likely to utilize CAT than those with inadequate knowledge and negative attitudes. These findings are consistent with previous evidence suggesting that awareness, positive beliefs, and perceived effectiveness are important determinants of CAT adoption and continued use (26, 27). Furthermore, multivariable logistic regression analysis confirmed that knowledge and attitudes remained independent predictors of CAT utilization after adjustment for socio-demographic characteristics. These findings highlight the important role of health literacy and personal beliefs in shaping healthcare decisions and treatment-seeking behaviors and further support the applicability of the Health Belief Model in understanding CAT utilization.

Several socio-demographic characteristics were significantly associated with CAT utilization. Occupation emerged as an important predictor, with self-employed and retired participants reporting the highest utilization rates. Similar observations have been reported by Ventola (28), who suggested that occupational status influences healthcare choices through differences in financial resources, healthcare needs, and access to information. Retired individuals may be particularly inclined to use CAT because of chronic health conditions, longer disease duration, and greater exposure to traditional healthcare practices accumulated over time.

Monthly income was also significantly associated with CAT utilization, with participants reporting adequate income demonstrating higher utilization rates than those with inadequate income. This finding agrees with previous research indicating that financial capability influences healthcare-seeking behavior and access to different treatment options (29, 30). Individuals with greater financial resources may have more opportunities to explore complementary therapies and purchase CAT-related products and services.

Marital status was another significant correlate of CAT utilization. Married and widowed participants reported higher utilization rates than single individuals. Similar findings have been reported in previous studies suggesting that family support systems, shared health beliefs, and social networks influence healthcare decisions and treatment choices (18–29). Married individuals may benefit from greater exposure to health-related discussions and recommendations within family environments, thereby increasing the likelihood of CAT adoption.

Educational level was significantly associated with both CAT utilization and knowledge level. Participants with diploma, university, and postgraduate qualifications demonstrated greater knowledge and utilization of CAT compared with individuals with lower educational attainment. These findings are consistent with studies conducted by Ahmad et al. (14) and Khan et al. (2), which identified education as an important determinant of awareness and acceptance of CAT. Higher educational attainment may improve health literacy, facilitate access to reliable health information, and enhance individuals’ ability to evaluate different healthcare options. Nevertheless, this finding is not universally supported. Some international studies have suggested that cultural traditions, family influences, and social norms may exert a stronger influence on CAT utilization than formal educational attainment alone (25, 26). In these contexts, individuals may adopt CAT practices based on inherited beliefs and community experiences regardless of their educational background. Such discrepancies may be explained by differences in healthcare systems, cultural environments, availability of CAT services, and variations in the populations under investigation.

Interestingly, no significant associations were observed between CAT utilization and either gender or nationality. This finding contrasts with several previous studies that reported greater utilization among females (14–27). One possible explanation is the widespread accessibility and cultural normalization of CAT practices within Saudi society, resulting in relatively similar utilization patterns across demographic groups. The absence of a significant association with nationality should be interpreted cautiously, given the overwhelming predominance of Saudi participants in the current sample.

Overall, the findings indicate that CAT is widely utilized and positively perceived among adults in the Qassim Region. Although participants demonstrated generally adequate knowledge and favorable attitudes toward CAT, important gaps remain regarding awareness of potential adverse effects and evidence-based use. These findings emphasize the need for public health initiatives aimed at improving health literacy, promoting safe CAT practices, strengthening communication between healthcare providers and CAT users, and supporting evidence-based integration of CAT within the Saudi healthcare system.

The present study demonstrated a significant association between participants’ knowledge level and CAT utilization. Individuals with adequate knowledge were significantly more likely to utilize complementary and alternative therapies than those with inadequate knowledge. This finding suggests that awareness and understanding of CAT play an important role in shaping health-related decisions and treatment-seeking behaviors. Similar findings have been reported in previous studies, which indicated that individuals with greater knowledge regarding CAT are more likely to explore and adopt complementary healthcare approaches (1, 5, 6). Higher knowledge levels may increase confidence in the use of CAT and improve individuals’ ability to identify appropriate therapies and access relevant information. However, despite the generally adequate knowledge observed in the present study, gaps remain regarding awareness of potential side effects and evidence-based use of CAT. Therefore, enhancing public education regarding both the benefits and risks of CAT remains essential to support informed healthcare decisions and promote safe utilization practices.

The findings further revealed a significant association between participants’ attitudes toward CAT and its utilization. Participants with positive attitudes were more likely to report CAT use than those with negative attitudes. This finding is consistent with previous research demonstrating that favorable beliefs regarding the effectiveness, safety, and value of CAT are important determinants of utilization (10, 11, 15). Individuals who perceive CAT as beneficial and compatible with their health beliefs are more likely to incorporate these therapies into their healthcare practices. The high proportion of participants demonstrating positive attitudes toward CAT (70.1%) may therefore explain the relatively high utilization rate observed in the present study. These findings are also consistent with the Health Belief Model, which suggests that individuals’ perceptions and beliefs significantly influence their health behaviors and decisions regarding treatment options.

Multivariable logistic regression analysis further confirmed that both knowledge and attitudes were independent predictors of CAT utilization after controlling for socio-demographic characteristics. Participants with adequate knowledge and positive attitudes demonstrated significantly higher odds of utilizing CAT than their counterparts. These findings indicate that the relationship between knowledge, attitudes, and utilization extends beyond simple demographic influences and reflects the important role of health literacy and personal perceptions in shaping healthcare choices. Similar findings have been reported in previous studies that identified knowledge and positive beliefs as key predictors of CAT adoption and continued use (1, 11, 28, 29). The persistence of these associations after adjustment for occupation, income, marital status, and educational level highlights the importance of educational interventions aimed at improving public understanding of CAT and promoting evidence-based decision-making.

In addition to knowledge and attitudes, the regression model identified occupation, monthly income, marital status, and educational level as significant independent predictors of CAT utilization. Participants who were self-employed, retired, married, and those with higher educational attainment were more likely to report CAT use. These findings support previous evidence suggesting that socio-economic and educational factors influence access to health information, healthcare resources, and health-seeking behaviors (15, 17, 28, 29). Individuals with higher educational levels may possess greater health literacy and a stronger ability to evaluate different healthcare options, while those with adequate financial resources may have increased access to complementary therapies. Collectively, these findings emphasize that CAT utilization is influenced by a complex interaction of demographic, cognitive, and attitudinal factors.

### Study limitations

Several limitations should be considered when interpreting the findings of this study. First, the cross-sectional design precludes establishing causal relationships. Second, the use of a self-administered online questionnaire may have introduced recall, reporting, and social desirability biases. Third, convenience sampling through online recruitment may have resulted in selection bias by overrepresenting individuals with internet access, higher digital literacy, or greater interest in CAT, thereby limiting the generalizability of the findings despite the relatively large sample size. Finally, the study did not assess the frequency, duration, effectiveness, or clinical outcomes of specific CAT modalities. Future studies using probability sampling and longitudinal designs are recommended to strengthen the evidence.

## Conclusion

The present study demonstrated that complementary and alternative therapies (CAT) are widely utilized among adults in the Qassim Region, Saudi Arabia, with more than half of the participants reporting previous or current CAT use. The findings revealed generally adequate knowledge and positive attitudes toward CAT, highlighting its growing acceptance as a complementary approach to healthcare. Nevertheless, important knowledge gaps remain, particularly regarding the potential side effects and evidence-based use of CAT. The study identified significant associations between CAT utilization and several socio-demographic characteristics, including occupation, monthly income, marital status, and educational level. Furthermore, adequate knowledge and positive attitudes toward CAT were significantly associated with higher utilization rates and remained independent predictors of CAT utilization after adjustment for demographic factors. These findings suggest that both socio-demographic characteristics and psychosocial factors play important roles in shaping public perceptions and utilization of CAT. Promoting evidence-based awareness, improving health literacy, and encouraging informed decision-making regarding CAT use are essential to ensure its safe and effective integration within healthcare practices. Public health initiatives and educational interventions should focus on enhancing awareness of both the benefits and potential risks of CAT while supporting effective communication between healthcare providers and CAT users. Such efforts may contribute to the safe incorporation of CAT within integrative healthcare models and improve overall healthcare outcomes.

### Study implications

The findings of this study have important implications for public health practice, healthcare providers, policymakers, and researchers. Public health authorities should develop targeted educational campaigns to improve awareness of the benefits, risks, and potential side effects associated with CAT, particularly among population groups with lower knowledge levels. Healthcare professionals should routinely assess patients’ use of CAT and provide evidence-based guidance to promote safe utilization and prevent potential adverse effects or interactions with conventional treatments. Policymakers should strengthen regulatory oversight of CAT products and practices to ensure safety, quality, and accurate public information. Furthermore, the positive attitudes observed toward CAT support the development of integrative healthcare models that facilitate communication between conventional healthcare providers and CAT users. Future research should investigate the effectiveness, safety, and long-term outcomes of commonly used CAT modalities through longitudinal and intervention-based studies to strengthen the evidence base for their integration into healthcare systems.

## Data Availability

The original contributions presented in the study are included in the article/Supplementary material, further inquiries can be directed to the corresponding author/s.

## References

[1] Al-Batanony MA Almutairi FF Almutairi AH Alrashodi AI Alsaif HM Alrashidi TA . Healthcare professionals' perception and practice of complementary and alternative medicine in Qassim region, Saudi Arabia. J Infect Dev Ctries. (2023) 17:1782–90. doi: 10.3855/jidc.18068, 38252731

[2] Khan A Ahmed ME Aldarmahi A Zaidi SF Subahi AM Al Shaikh A . Awareness, self-use, perceptions, beliefs, and attitudes toward complementary and alternative medicines (CAM) among health professional students in king Saud bin Abdulaziz University for Health Sciences Jeddah, Saudi Arabia. Evid Based Complement Alternat Med. (2020) 2020:1–11. doi: 10.1155/2020/7872819 PMC7191386 32382305

[3] Avila C Grace S Bradbury J. How do patients integrate complementary medicine with mainstream healthcare? A survey of patients’ perspectives. Complement Ther Med. (2020) 49:102317. doi: 10.1016/j.ctim.2020.102317, 32147079

[4] Zaidi SF Alzahrani A Alghamdy Z Alnajar D Alsubhi N Khan A . Use of complementary and alternative medicine in the general public of Western Saudi Arabia: a cross-sectional survey. Cureus. (2022) 14:e32784. doi: 10.7759/cureus.32784, 36570109 PMC9772711

[5] Bhuyan DJ Dissanayake I Jaye K Chang D. Traditional and complementary medicine in Australia: clinical practice, research, education, and regulation. Int J Ayurveda Res. (2022) 3:16–29. doi: 10.4103/ijar.ijar_4_22

[6] Al Mansour MA Mohamed EY Abdalla SM Medani KA Mahmoud WS Meraj SA. Satisfaction, self-use and perception of medical students in Majmaah University, Kingdom of Saudi Arabia, towards complementary and alternative medicine. J Taibah Univ Med Sci. (2015) 10:74–8. doi: 10.1016/j.jtumed.2015.01.009, 42574925

[7] Jaiswal K Bajait C Pimpalkhute S Sontakke S Dakhale G Magdum A. Knowledge, attitude and practice of complementary and alternative medicine: a patient′s perspective. Int J Med Public Health. (2015) 5:19. doi: 10.4103/2230-8598.151243

[8] Dehghan M Rad MM Lari LA Ghorbani-nejad B Mohebi-Rad M. The relationship between use of complementary and alternative medicine and health literacy in chronically ill outpatient cases: a cross-sectional study in southeastern Iran. Front Public Health. (2023) 11:388. doi: 10.3389/fpubh.2023.988388 PMC10173769 37181699

[9] Belachew N Tadesse T Gube AA. Knowledge, attitude, and practice of complementary and alternative medicine among residents of Wayu town, Western Ethiopia. J Evid Based Complement Altern Med. (2017) 22:929–35. doi: 10.1177/2515690X17746547, 29250965 PMC5871321

[10] Khan A Ahmed ME Aldarmahi A Al Shaikh A Feroz Z Rehman D-e-S . Awareness, perceptions and attitudes towards complementary and alternative medicine in Saudi Arabia: a cross-sectional and comparative study of students of healthcare professions. Trop J Pharm Res. (2022) 20:1307–19. doi: 10.4314/tjpr.v20i6.30

[11] Cortés-Ladino AC Arias-Ortiz AW Porras-Ramírez A. Effectiveness of yoga and acupuncture in rheumatoid arthritis: a systematic review and Meta-analysis. Evid Based Complement Altern Med. (2023) 2023:1–10. doi: 10.1155/2023/9098442 PMC10575744 37842334

[12] Elfaki BA. Perspectives on complementary and alternative medicine: the role of nurses and health care providers: a review. J Posit Sch Psychol. (2022) 2022:5978–84.

[13] Bello N Winit-Watjana W Baquir W McGarry K. Disclosure and adverse effects of complementary and alternative medicine used by hospitalized patients in the north east of England. Pharm Pract. (2012) 10:125–35. doi: 10.4321/s1886-36552012000300002, 24155828 PMC3780488

[14] Ahmad W Ahmad A Hassan Y Hassan YA Sivapalan N Daniel S . Awareness, knowledge, attitude, perception, and utilization of complementary and alternative medicines (CAMs) in the common population of Dammam, Saudi Arabia. J Pharm Bioallied Sci. (2022) 14:99–105. doi: 10.4103/jpbs.jpbs_186_22, 36034491 PMC9416104

[15] Posadzki P Watson LK Alotaibi A Ernst E. Prevalence of use of complementary and alternative medicine (CAM) by patients/consumers in the UK: systematic review of surveys. Clin Med (Northfield Il). (2013) 13:126–31. doi: 10.7861/clinmedicine.13-2-126, 23681857 PMC4952625

[16] Ernst E. Prevalence of use of complementry/alternative medicine: a systematic review. Bull World Health Organ. (2000) 78:252–7. PMC2560678 10743298

[17] Harris PE Cooper KL Relton C Thomas KJ. Prevalence of complementary and alternative medicine (CAM) use by the general population: a systematic review and update. Int J Clin Pract. (2012) 66:924–39. doi: 10.1111/j.1742-1241.2012.02945.x, 22994327

[18] Alrowais NA Alyousefi NA. The prevalence extent of complementary and alternative medicine (CAM) use among Saudis. Saudi Pharm J. (2017) 25:306–18. doi: 10.1016/j.jsps.2016.09.009, 28344484 PMC5357106

[19] Ashraf M Saeed H Saleem Z Rathore HA Rasool F Tahir E . A cross-sectional assessment of knowledge, attitudes and self-perceived effectiveness of complementary and alternative medicine among pharmacy and non-pharmacy university students. BMC Complement Altern Med. (2019) 19:95. doi: 10.1186/s12906-019-2503-y, 31053114 PMC6500055

[20] Awad AI Al-Ajmi S Waheedi MA. Knowledge, perceptions and attitudes toward complementary and alternative therapies among Kuwaiti medical and pharmacy students. Med Princ Pract. (2012) 21:350–4. doi: 10.1159/000336216, 22377503

[21] James PB Bah AJ. Awareness, use, attitude and perceived need for complementary and alternative medicine (CAM) education among undergraduate pharmacy students in Sierra Leone: a descriptive cross-sectional survey. BMC Complement Altern Med. (2014) 14:438. doi: 10.1186/1472-6882-14-438, 25380656 PMC4236455

[22] Ameade EPK Amalba A Helegbe GK Mohammed BS. Medical students’ knowledge and attitude towards complementary and alternative medicine – a survey in Ghana. J Tradit Complement Med. (2016) 6:230–6. doi: 10.1016/j.jtcme.2015.03.004, 27419086 PMC4936753

[23] Pokladnikova J Lie D. Comparison of attitudes, beliefs, and resource-seeking behavior for CAM among first- and third-year Czech pharmacy students. Am J Pharm Educ. (2008) 72:24. doi: 10.5688/aj720224, 18483592 PMC2384199

[24] Ng JY Verhoeff N Steen J. What are the ways in which social media is used in the context of complementary and alternative medicine in the health and medical scholarly literature? A scoping review. BMC Complement Med Ther. (2023) 23:32. doi: 10.1186/s12906-023-03856-6, 36732809 PMC9893203

[25] Siedlecki SL. Complementary and alternative therapies (CAT) in academic programs and nursing practice: is more education is needed? Complement Ther Clin Pract. (2021) 43:101327. doi: 10.1016/j.ctcp.2021.101327, 33550192

[26] Abubakar U Zulkarnain AI Samri F Hisham SR Alias A Ishak M . Use of complementary and alternative therapies for the treatment of dysmenorrhea among undergraduate pharmacy students in Malaysia: a cross sectional study. BMC Complement Med Ther. (2020) 20:285. doi: 10.1186/s12906-020-03082-4, 32948163 PMC7501717

[27] Wells RE Bertisch SM Buettner C Phillips RS McCarthy EP. Complementary and alternative medicine use among adults with migraines/severe headaches. Headache J Head Face Pain. (2011) 51:1087–97. doi: 10.1111/j.1526-4610.2011.01917.x, 21649654 PMC3627391

[28] Ventola CL. Current issues regarding complementary and alternative medicine (CAM) in the United States: part 1: the widespread use of CAM and the need for better-informed health care professionals to provide patient counseling. P T. (2010) 35:461–8. 20844696 PMC2935644

[29] Latunji OO Akinyemi OO. Factors influencing health-seeking behaviour among civil servants in Ibadan, Nigeria. Ann Ibadan Postgrad Med. (2018) 16:52–60. PMC6143883 30254559

[30] Gyasi RM Adam AM Phillips DR. Financial inclusion, health-seeking behavior, and health outcomes among older adults in Ghana. Res Aging. (2019) 41:794–820. doi: 10.1177/0164027519846604, 31046598

